# Total Phenolic Contents and Antioxidant Potential of Herbs Used for Medical and Culinary Purposes

**DOI:** 10.1007/s11130-018-0699-5

**Published:** 2018-10-29

**Authors:** Beata Ulewicz-Magulska, Marek Wesolowski

**Affiliations:** Department of Analytical Chemistry, Medical University of Gdansk, Gen. J. Hallera 107, 80-416 Gdansk, Poland

**Keywords:** Total phenolic content, Total antioxidant capacity, Medicinal herbs, Spices

## Abstract

Herbs used for medical purposes are required to meet high pharmacopoeial quality standards, whereas spices used as additives to dishes and food products do not have to meet such rigorous standards. Therefore, the aim of this paper was to compare medicinal herbs and spices of the same plant species that are applied in different areas with regard to the total phenolic content (TPC) and total antioxidant capacity (TAC) of their methanolic and water extracts. This study showed that the TPC values of both extracts prepared from medicinal herbs and spices as quantified using the Folin-Ciocalteu reagent did not differ significantly; however, the TAC values obtained by the DPPH assays of the methanol and water extracts differed significantly. No such differences were found for the same extracts when the FRAP assay was used for analysis. This study shows that the level of antioxidants is dependent on the plant species and the botanical family. Plants belonging to the Lamiaceae and Asteraceae families are richer in phenolic compounds and have stronger antioxidant potentials than those originating from the Apiaceae family. Multivariate statistical analysis indicates that the majority of medicinal herbs and spices of the same plant species are found in the same cluster. In addition, the extraction solvent was identified as one of the factors influencing the clustering of the plants on the PCA scatterplot and CA dendrogram.

**Electronic supplementary material:**

The online version of this article (10.1007/s11130-018-0699-5) contains supplementary material, which is available to authorized users.

## Introduction

The human body possesses innate defense mechanisms, such as superoxide dismutase, glutathione peroxidase, catalase, glutathione, ubiquinone and uric acid, to neutralize free radicals in the form of endogenous antioxidants [1, 2]. However, the quantities of these defenders generated in the body are likely to be inadequate, especially under conditions of oxidative stress or inflammation during which the production of free radicals is increased. Hence, plants, as a rich source of natural antioxidants, can complement endogenous antioxidant systems to a point where the levels are sufficient [2]. While natural antioxidants occur in all parts of all higher plants, those with medicinal or culinary uses are valuable sources of antioxidants, such as vitamins A, E and C and phenolic compounds including phenolic acids, flavonoids, lignin, stilbenes and tannins [3, 4]. These secondary metabolites also exhibit anti-inflammatory, antibacterial, antiviral, anticancer and immune-stimulating activities [5].

Antioxidants are also used in industry in order to prolong the stability of foods and cosmetics [6] with synthetic antioxidants such as propyl gallate, butylated hydroxy-anisole (BHA) and butylated hydroxytoluene (BHT) being particularly common. However, the use of these antioxidants has been questioned due to their potential health risks and toxicity [6, 7]. Thus, the search for antioxidants from natural sources, such as aromatic spice plants, is currently attracting much attention, not only because of the scavenging properties of these compounds but also because they are natural, nonsynthetic products, which is highly appreciated by consumers [8]. Some of these plants have been studied numerous times, which has resulted in the development of natural formulations for inactivating free radicals. Because information on the antioxidant properties of some plants, particularly those less widely used for medical and culinary purposes, is still rather limited, the assessment of these properties remains an interesting and useful task [5, 8–10]. The aim of this study was therefore to compare these parameters in common herbs used for medicinal and culinary purposes.

Medicinal herbs and spices were chosen for this study because of the significant difference in quality requirements between the two areas of application. Pharmaceutical law demands that herbs used for medical purposes meet appropriate quality standards [11], whereas spices applied as additives to dishes and food products for improving flavor or prolonging stability are not so rigorously controlled. Hence, it is interesting to compare the total phenolic content (TPC) and total antioxidant capacity (TAC) of medicinal herbs and spices of the same plant species but from different areas of application. The aim of this work was realized by quantitation of TPC in the methanolic and water extracts of medicinal herbs and spices using the Folin-Ciocalteu reagent and determining their free radical scavenging activities with the DPPH assay and their ferric reducing antioxidant power (FRAP) with the corresponding assay. Two tests commonly used for assessment of TAC of plants, their extracts and plant-derived foods were chosen due to the complex phytochemical composition of the plants studies herein [5, 12, 13].

## Materials and Methods

### Plant Material

A set of 21 samples consisting of 10 medicinal herbs and 11 spices, all of which were obtained in powdered form, was used for analysis. There was no information on the packaging labels about the standardization of some of the active constituents in the medicinal herbs. Three to ten independent samples of each item were analyzed (Table 1); thus, a total set of 121 samples, including 55 medicinal herb samples and 66 spice samples, was examined. The distributors of these samples were herbal enterprises in Poland – Dar Natury (Koryciny), Sigal (Wierzchoslawice), Kamis (Stefanowo), Prymat (Jastrzebie Zdroj), Kawon (Gostyn), Dobra (Suchy Las), Kotanyi (Warszawa), and Flos (Mokrsko). The samples were homogenized at 20 °C for 20 s in a water-cooled grinder Knifetec 1095 (Foss Tecator, Höganäs, Sweden) and kept in closed containers to protect against daylight and moisture until analysis.

**Table 1:** Total phenolic contents (TPC) and total antioxidant capacity for methanolic and water extracts of herbs and spices determined using DPPH test (TAC_DPPH_) and FRAP test (TAC_FRAP_)

Sample number	Plants	Methanolic extracts			Water extracts		
		TPC [mg GA/g]	TAC_DPPH_ [%]	TAC_FRAP_ [mmol Fe ^2+^/g]	TPC [mg GA/g]	TAC_DPPH_ [%]	TAC_FRAP_ [mmol Fe ^2+^/g]
Medicinal herbs from Lamiaceae family							
1	Rosemary leaves (*n* = 6)	51.2–178.8 106.2 (125.3)	41.6–64.7 52.4 (53.5)	2.2–6.5 4.3 (4.4)	50.0–96.5 77.2 (80.8)	19.8–45.5 35.0 (36.8)	4.2–10.3 6.8 (6.9)
2	Sage leaves (*n* = 9)	16.2–173.6 61.3 (39.2)	14.1–64.5 36.23 (34.22)	2.2–7.3 5.1 (5.1)	25.7–91.6 57.4 (54.1)	6.4–48.5 26.0 (25.3)	3.2–7.9 5.5 (5.4)
3	Thyme herbs (*n* = 5)	42.4–240.8 125.9 (93.6)	32.5–79.3 54.56 (50.36)	0.6–15.4 6.7 (1.7)	42.9–99.6 75.2 (75.6)	12.4–57.6 35.0 (38.3)	3.8–13.6 7.9 (7.5)
4	Oregano herbs (*n* = 3)	104.3–325.1 214.6 (214.2)	46.8–87.2 70.4 (77.7)	6.8–12.4 9.4 (9.2)	109.8–306.7 219.1 (289.1)	28.6–61.1 48.5 (55.8)	21.5–22.3 22.0 (22.1)
5	Basil herbs (*n* = 3)	28.2–29.9 29.0 (29.0)	19.9–28.3 24.1 (24.1)	0.9–1.6 1.3 (1.2)	44.4–47.5 46.0 (46.0)	13.9–18.8 16.4 (16.4)	1.2–2.4 1.8 (1.4)
6	Melissa leaves (*n* = 8)	54.9–299.5 124.61 (88.71)	32.1–87.5 54.4 (51.6)	9.7–18.3 14.9 (15.9)	87.9–204.8 132.4 (109.4)	25.7–67.2 48.7 (48.1)	20.5–26.9 23.9 (24.0)
7	Peppermint leaves (*n* = 9)	18.3–284.3 85.6(41.3)	14.5–76.2 38.4 (39.9)	4.1–14.7 8.7 (7.5)	40.9–185.6 81.6 (70.6)	11.5–61.7 32.8 (28.6)	9.5–18.7 15.1 (17.5)
Medicinal herbs from Apiaceae family							
8	Caraway seed (*n* = 3)	8.9–26.9 14.4 (13.5)	1.3–21.1 9.56(5.5)	0.1–0.2 0.1 (0.1)	10.0–24.8 15.9 (15.9)	4.20–2.6 11.2 (6.1)	0.1–0.2 0.2 (0.2)
9	Lovage roots (*n* = 4)	13.8–26.4 17.0 (15.6)	2.9–22.7 9.1 (6.1)	0.2–0.4 0.4 (0.4)	10.7–19.8 14.208 (14.2)	1.8–22.4 8.1 (4.1)	0.2–0.2 0.2 (0.2)
10	Angelica roots (*n* = 5)	10.9–19.2 15.2 (15.2)	1.7–21.6 10.5 (7.4)	0.1–0.2 0.1 (0.2)	10.2–21.6 15.6 (15.4)	1.9–22.8 11.5 (6.9)	0.1–0.2 0.2 (0.22)
Spices from Lamiaceae family							
11	Rosemary (*n* = 4)	57.2–147.7 90.9 (74.2)	43.2–65.9 51.9 (48.2)	4.0–4.4 4.2 (4.2)	56.8–87.67 70.2 (69.3)	19.3–43.4 29.6 (26.6)	1.7–3.1 2.5 (2.6)
12	Sage (*n* = 3)	103.2–134.9 114.9 (106.5)	53.2–61.0 57.9 (58.8)	4.8–5.67 5.1 (5.0)	71.6–75.0 72.9 (72.6)	24.5–34.8 29.6 (29.5)	2.3–3.1 2.7 (2.8)
13	Thyme (*n* = 5)	41.2–212.4 112.6 (72.0)	28.5–69.6 50.2 (46.9)	0.8–3.0 1.9 (1.8)	50.1–163.8 86.7 (73.8)	14.3–42.9 27.2 (24.1)	2.2–6.9 4.7 (4.8)
14	Oregano (*n* = 9)	48.0–192.0 105.5 (108.4)	32.1–65.6 46.6 (45.4)	2.9–6.1 4.6 (5.0)	45.6–91.7 70.8 (73.2)	16.1–43.9 28.6 (28.0)	1.8–4.3 2.9 (3.0)
15	Basil (*n* = 10)	31.7–112.6 58.8 (59.3)	16.5–36.0 26.6 (26.3)	0.6–4.7 2.3 (2.2)	36.7–78.4 53.4 (49.3)	11.6–37.6 25.5 (28.1)	1.2–3.1 2.3 (2.41)
16	Marjoram (*n* = 9)	51.8–236.0 118.4 (130.9)	19.3–53.04 41.6 (44.3)	3.2–7.2 5.0 (4.8)	53.0–190.9 79.2 (68.3)	6.9–41.4 25.9 (28.2)	1.8–7.1 3.1 (2.5)
17	Savory (*n* = 4)	38.4–140.2 95.0 (108.0)	26.2–47.5 37.3 (36.6)	1.6–4.3 2.7 (2.6)	55.2–74.2 64.9 (65.3)	13.0–33.5 24.8 (24.2)	2.1–3.2 2.6 (2.5)
18	Hyssop (*n* = 4)	18.3–47.1 35.5 (39.1)	9.8–51.7 29.1 (31.5)	1.6–2.8 2.2 (2.1)	28.7–136.3 81.3 (84.5)	5.1–32.4 21.0 (22.9)	4.4–5.7 4.89 (4.8)
Spices from Apiaceae family							
19	Caraway (*n* = 8)	8.7–17.3 12.2 (11.7)	3.9–19.5 11.2 (12.5)	0.1–0.2 0.15(0.2)	6.4–21.5 12.6 (9.7)	2.0–28.6 15.2 (20.6)	0.2–0.2 0.2 (0.2)
20	Lovage (*n* = 6)	12.9–73.8 36.4 (27.8)	4.8–21.1 12.5 (12.4)	0.5–1.8 1.1 (1.0)	20.0–50.7 33.9 (33.1)	3.6–30.5 14.2 (8.7)	0.5–1.3 0.8 (0.8)
Spices from Asteraceae family							
21	Tarragon (*n* = 4)	97.2–253.5 151.6 (134.6)	29.0–64.7 40.4 (35.2)	2.7–4.3 3.2 (3.4)	59.5–198.3 102.8 (82.6)	25.6–45.8 32.5 (30.7)	1.9–6.3 3.4 (3.8)

The results are expressed as the range, arithmetical mean and median in the parenthesis

*n* number of independent samples, each being analysed at least in triplicate

### Analytical Methods

Methanol (80%) and deionized water were used to prepare extracts based on the procedures reported by Wong et al. [14] and Wojdyło et al. [5]. The TPC was determined using Folin-Ciocalteu reagent according to the procedure described by Hinneburg et al. [15]. The results are expressed as milligrams of gallic acid equivalents per gram dry plant weight (mg GA/g). The TAC of the extracts was determined by the DPPH assay with a synthetic radical, 2,2-diphenyl-1-picrylhydrazyl (DPPH), and by a FRAP assay. The former assay evaluates the ability of antioxidants to act as free radical scavengers or hydrogen donors [16, 17], whereas the latter is an indicator of the reducing power of the antioxidant, *i.e*., in reducing the ferric ions to the ferrous ions [14, 18]. The DPPH values of the extracts are expressed in percentages, while the FRAP values are expressed in mmol of Fe^2+^ per gram dry plant weight (mmol Fe^2+^/g).

### Statistical Calculations

Statistical calculations using the Kolmogorov-Smirnov test, Pearson’s correlation analysis, principal component analysis and cluster analysis were done by Statistica software, ver. 10 (StatSoft, Tulsa, OK, USA).

## Results and Discussion

Previous studies (Table 1, Supplementary Materials) have confirmed that medicinal herbs and spices are abundant in essential oils and phenolic compounds, such as phenolic acids, flavonoids and flavonoid derivatives. Some of these plants also contained bitter diterpenic compounds and triterpenic acids, but others contained tannins, carbohydrates, steroids or lipids. As shown in Table 1, the means of the TPC and TAC values from the methanolic and water extracts show that the TPC values in the methanolic extracts vary over the ranges of 14.4–214.6 and 12.2–151.6 mg GA/g for medicinal herbs and spices, respectively, and these data were similar to those from the water extracts; 14.2–219.1 and 12.6–102.8 mg GA/g for the same herbs and spices, respectively. A Kolmogorov-Smirnov test showed that the TPCs in the extracts prepared from herbs and spices did not significantly differ (*p* > 0.1, α = 0.05). Changing extraction solvents (methanolic and water extracts) also had no significant impact on the total phenolic. Similar values for the TPC (6.4–180.5 mg GA/g) were determined for Thai plants (extracts with 95% ethanol) [19], traditional Chinese medicinal plants (1.1–52.3 mg GA/g in extracts with 80% methanol) [7], and culinary herbs and spices from Finland (18.5–147.0 mg GA/g in water extracts) [15].

Plants belonging to the Lamiaceae family, *i.e*., *Rosmarinus officinalis*, *Salvia officinalis*, *Thymus vulgaris*, *Origanum vulgare*, *Ocimum basilicum*, *Melissa officinalis*, *Mentha piperita*, *Origanum majorana*, *Satureja hortensis* and *Hyssopus officinalis* are characterized by high levels of phenolic compounds, and in many cases, the contents of these compounds exceeds 100 mg GA/g. Among medicinal plants, those with the highest contents of phenolic compounds were the herbs oregano and thyme as well as the leaves of melissa and rosemary. Among spices, the highest TPC values were found in marjoram, sage, thyme, oregano and tarragon, and the latter belongs to the Asteraeae family. On the other hand, plants belonging to the Apiaceae family, *Carum carvi*, *Archangelica officinalis* and *Levisticum officinalis*, were characterized by very low TPC values; their TPC values were approximately 12 mg GA/g. Lovage was found to be an exception; the leaves, which are used in the dried or powdered form for culinary purposes, are characterized by higher concentrations of phenolic compounds (36.42 and 33.95 mg GA/g in the methanolic and water extracts, respectively) than the dried roots that are used for medicinal purposes (16.99 and 14.20 mg GA/g in the methanolic and water extracts, respectively).

As shown in Table 1, the antioxidant capacities determined using the DPPH assay vary substantially, *i.e*., 9.1–70.4% and 8.1–48.7% for the methanolic and water extracts, respectively. In the case of the FRAP assay, values ranging from 0.1–14.9 and 0.2–23.9 mmol Fe^2+^/g were found for the methanolic and water extracts, respectively. The TAC values obtained from the DPPH assay for the methanol and water extracts differ significantly (*p* < 0.01, α = 0.05), whereas no significant differences were found for the same extracts in the case of the FRAP assay (*p* > 0.1, α = 0.05). This indicates that the difference between the antioxidant activities of the methanolic and water extracts is due to the assay used rather than the extraction solvent. DPPH is only soluble in organic media, which is an important limitation in the determination of hydrophilic antioxidants. Moreover, the reaction of DPPH with antioxidants also depends on the chemical structure of the antioxidants [5, 20]. The results obtained using the FRAP assay for the alcoholic and water extracts are comparable [7].

The highest TAC values expressed as the free radical scavenging activity (DPPH assay) were shown by the methanolic extracts prepared from plant species belonging to the Lamiaceae family. Their activities can be arranged in the following order: oregano herbs (TAC above 70%) > sage > thyme herbs > melissa leaves > rosemary leaves > rosemary > thyme (TAC above 50%) > oregano > marjoram > peppermint leaves > sage leaves > savory > hyssop > basil > basil herbs. Similarly, most of the water extracts of the medicinal herbs and spices originating from the Lamiaceae family are also characterized by high antioxidant potentials (above 20–50% DPPH radical activity inhibition). This strong antioxidant potential may be due to rosmarinic acid, a plant metabolite with a pronounced ability to inhibit DPPH radical activity. Plants such as *Origanum vulgare*, *Mentha piperita*, *Melissa officinalis*, *Rosmarinus officinalis* and *Thymus vulgaris* are especially rich in this acid. Other phenolic acids, *e.g*., caffeic, chlorogenic and ferulic acids, also play a part in neutralizing free radicals [4]. Very low quantities of phenolic compounds are found in the roots of lovage and angelica, as well as in caraway seeds, *i.e*., medicinal herbs belonging to the Apiaceae family, and the antioxidant capacities of these plant materials were found to be 8–12%.

The FRAP assay shows that the highest TAC values, expressed as ferric reducing antioxidant power, were displayed by the methanolic and water extracts of medicinal plants, *i.e*., the leaves of melissa and peppermint and the herb oregano (in this case, the values were similar to that determined with the DPPH assay). In contrast, samples belonging to the Apiaceae family – the roots of lovage and angelica and caraway seeds have the lowest antioxidant activities (below 1 mmol Fe^2+^/g in both the methanolic and water extracts). All the spice extracts had low antioxidant capacities based on the FRAP assay (below 5 mmol Fe^2+^/g).

Comparison between the medicinal herbs and spices shows that the methanolic extract of oregano herb has a phenolic content and an antioxidant potential that are twice those of oregano spice. Much larger differences were found between the water extracts of this medicinal herb and spice. In addition, the mean content of the TPC and the DPPH value for methanolic extract of sage (spice) were higher than those for sage leaves. A similar tendency was also observed with the extracts of lovage (spice) and lovage roots; however, sample materials were obtained from different morphological parts of the plant (spice from the powdered leaves, medicinal herbs from the roots) with the same being true for basil (spice) and leaves.

Pearson’ correlation coefficients between the TPC and TAC values from the DPPH (TAC_DPPH_) and FRAP (TAC_FRAP_) assays revealed a strong relationship (*r* = 0.94) between the free radical scavenging activity (DPPH assay) and the ferric reducing antioxidant power (FRAP assay) of the water extracts, while the same relationship between the methanol extracts was found to be considerably weaker (*r* = 0.73). Significant correlations over the range of 0.70 and 0.87 were also observed between the TAC_DPPH_ and TAC_FRAP_ values for both extraction solvents. These results are consistent with those reported in another study of TAC_DPPH_ and TAC_FRAP_ values of plant extracts [1, 6, 13]. Furthermore, significant correlations were found among TAC_DPPH_ and TAC_FRAP_ and TPC for the methanolic and water extracts prepared from medicinal herbs and spices. These strong correlations confirmed that the antioxidants found in plants are capable of both free radical scavenging and antioxidant reduction [7].

The results of this study are consistent with those found in the literature, *e.g*., high correlations within the range of 0.7–0.9 were reported for TAC_DPPH_ and TPC values [1, 4, 13, 21], whereas correlations ranging between 0.87 and 0.98 were reported for the TAC_FRAP_ and TPC values in extracts from medicinal plants [1, 13, 22, 23]. These results show that medicinal herbs and spices with high total antioxidant capacities are characterized by high levels of phenolic compounds. The results for the herbs of oregano and thyme, the leaves of rosemary and melissa, and for the spices thyme, rosemary and sage, confirm these findings.

To elucidate the relationships between the medicinal herbs and spices regarding their antioxidant capacity, contents of phenolic compounds, plant species and botanic families as well as the kind of extractant used (methanol and water), two unsupervised techniques of advanced multivariate statistical analysis, principal component analysis (PCA) and cluster analysis (CA), were chosen [24].

**Fig. 1:**
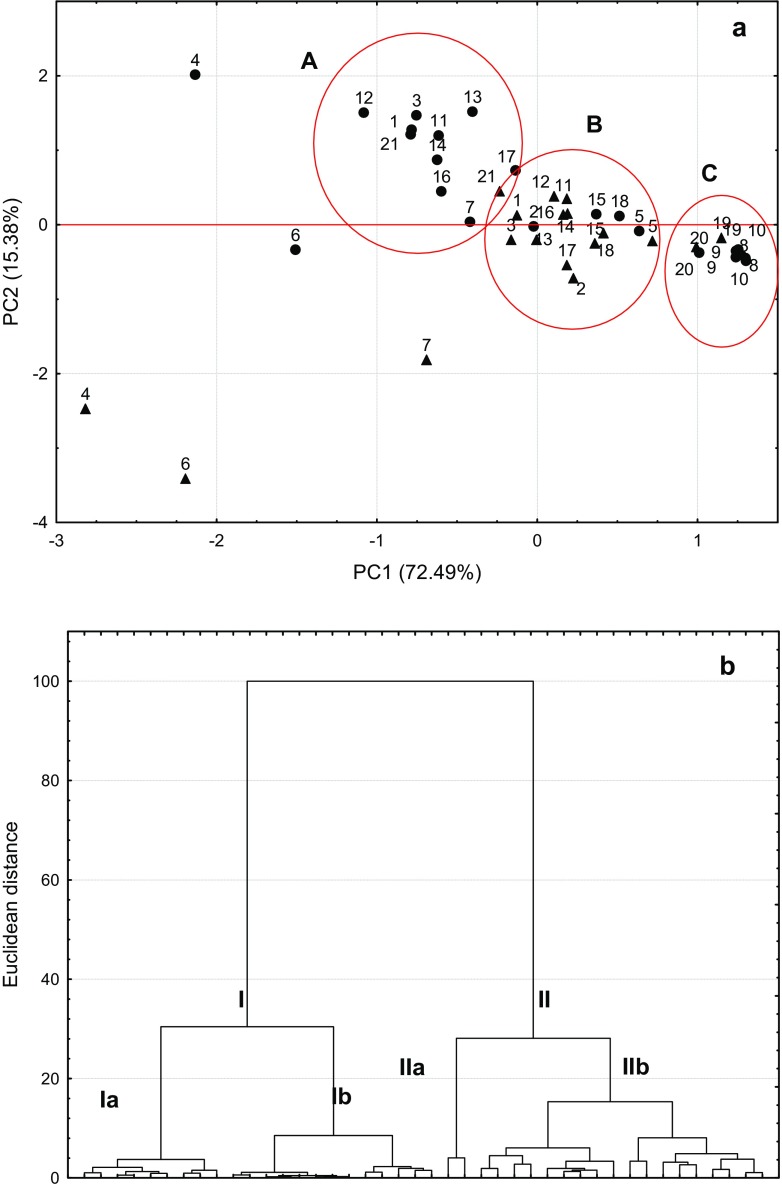
**a** PCA scatterplot for medicinal herbs and **b** CA dendrogram for medicinal herbs and spices. Herbs: rosemary leaves (1), sage leaves (2), thyme herbs (3), oregano herbs (4), basil herbs (5), melissa leaves (6), peppermint leaves (7), caraway seeds (8), lovage roots (9), and angelica roots (10); spices: rosemary (11), sage (12), thyme (13), oregano (14), basil (15), marjoram (16), savory (17), hyssop (18), caraway (19), lovage (20), and tarragon (21) ● – methanol extract, ▲ – water extract

Figure 1a shows that two first principal components (PC1 and PC2) explain more than 87% of the data variability, and a set of 21 samples consisting of 10 medicinal herbs and 11 spices were separated into three clusters (A, B and C). Among these clusters, both the methanolic and water extracts of oregano herbs and melissa leaves are located on the left-hand side of the PCA scatterplot. These medicinal herbs in the Lamiaceae family had the highest TPC and TAC values of all the plants studied. The majority of the remaining samples are grouped in clusters A and B based on their TPC and TAC data, and the extraction solvent affected the distribution of the samples. Cluster A includes methanolic extracts and notably the water extracts of plants rich in TPC that displayed strong antioxidant activities, such as the extracts of rosemary leaves and spice, thyme herb and spice, peppermint leaves, and the spices sage, oregano, marjoram and savory as well as methanolic and water extracts of tarragon (the Asteraceae family). The water extracts of plants were grouped in cluster B owing to their lower contents of phenolic compounds and weaker antioxidant activities compared to those of the methanolic extracts. Both extracts of sage leaves, basil herbs and spice, and hyssop spice were found in this cluster. In contrast, the plant samples with the lowest TPC values and demonstrating the lowest antioxidant activities are located on the right-hand side of the PCA scatterplot (cluster C). This cluster includes all the plants originating from the Apiaceae family, *i.e*., both the methanolic and water extracts of the medicinal herbs and spices of caraway, lovage and angelica. These findings were confirmed by the CA dendrogram (Fig. 1b), which indicates the same characteristic groups of plant samples. Subclusters Ia and Ib encompass the extracts of plants grouped in clusters A and C, respectively, on the PCA scatterplot, whereas cluster II consists of those samples located in cluster B on the PCA scatterplot.

## Conclusions

This study shows that the majority of medicinal herbs and spices have similar TPC and TAC levels. An exception is the methanolic extract of oregano herbs, which showed two-fold higher values of TPC and TAC than those of oregano spice. Much higher differences were also found for the water extracts. The opposite tendency was observed for lovage because the leaves, as a distinct morphological part of the plant, are generally richer in the secondary metabolites than the roots, which are used for medicinal purposes.

This study also demonstrates that the level of antioxidants, expressed in terms of the TPC, in these medical herbs and spices depends on the plant species and botanical family. Plants belonging to the Lamiaceae and Asteraceae families are richer in TPC and have stronger antioxidant potentials than those originating from the Apiaceae family. Oregano herbs and spice, thyme herbs and spice, rosemary leaves and spice and melissa leaves, as well as marjoram, sage, and tarragon (the Asteraceae family), were found to have the highest contents of phenolic compounds and the highest antioxidant activities. The Kolmogorov-Smirnov test revealed that the TAC values obtained by the DPPH assay for the methanolic and water extracts differ significantly, whereas no significant differences were found for the same extracts based on the FRAP assay. This result suggests that these differences are due to the assay used rather than the extraction solvent. The results of this study were verified by multivariate statistical analysis techniques, PCA and CA, which indicated that the majority of medicinal herbs and spices from the same plant species are found in the same cluster. Moreover, the type of extraction solvent was identified as one of the factors discriminating the plants on the PCA scatterplot and CA dendrogram.

## Electronic supplementary material

ESM 1 (DOCX 15 kb)

ESM 2 (DOCX 24 kb)

## Abbreviations

DPPH: 2,2-diphenyl-1-picrylhydrazyl
FRAP: ferric reducing antioxidant power
TAC: total antioxidant capacity
TPC: total phenolic content
