# Supplementary material for: Total Phenolic Contents and Antioxidant Potential of Herbs Used for Medical and Culinary Purposes
Source: Plant Foods Hum Nutr. 2018 Oct 29;74(1):61–7. doi: 10.1007/s11130-018-0699-5 (PMC6422988; doi:10.1007/s11130-018-0699-5)
Supplement: Supplementary file 1 — (DOCX 15 kb) [file 11130_2018_699_MOESM1_ESM.docx]

Total phenolic contents and antioxidant potential of herbs used for medical and culinary purposes. Plant Foods for Human Nutrition.

Beata Ulewicz-Magulska · Marek Wesolowski, Department of Analytical Chemistry, Medical University of Gdansk, Gen. J. Hallera 107, 80-416 Gdansk, Poland, *E-mail address*: [marwes@gumed.edu.pl](mailto:marwes@gumed.edu.pl)

Detailed procedures for extracts preparation, determination of total phenolic contents and assessment of total antioxidant capacity were as follows:

**Extracts Preparation**

The methanolic extracts were obtained by sonication of a 0.5-g sample of each plant with 4 mL of 80% methanol using an ultrasonic bath (model Sonic 6, Polsonic, Warsaw, Poland) for 30 min. After centrifugation at 20.000 rpm for 10 min with a centrifuge (EBA 20S, Hettich, Tuttlingen, Germany), the supernatant was removed by decanting. The sample was re-extracted for a total of three extractions with 4 mL of 80% methanol. The combined extracts were filtered through a paper filter (Macherey-Nagel, Düren, Germany) and kept in a refrigerator at +4°C until further analysis. For water extraction, a 0.5-g portion of finely powdered plant material was weighed into a beaker, and then 40 mL of redistilled water at ambient temperature was added. The extraction was carried out by heating under a watch glass in a water bath (LW-12, Cabrolab, Warsaw, Poland) and boiling for 1 h. After cooling, the extract was filtered through a paper filter and kept in refrigerator at +4°C until analysis. Redistilled water was obtained by triple distillation of water using a Destamat® Bi-18 system (Heraeus Quarzglas, Hanau, Germany).

**Determination of Total Phenolic Contents**

To a glass tube with 4 mL of redistilled water were added 1 mL of the diluted extract and 0.5 mL of Folin-Ciocalteu reagent. After 2 min, 1 mL of a 20% solution of sodium carbonate was added, and the solution was diluted to 10 mL with redistilled water. Then, the tube was vortexed for 10 s and left to stand in the dark for 30 min. The absorbance of the solution was measured at 760 nm by a UV-1202 spectrometer (Shimadzu, Duisburg, Germany) against a blank.

**Assessment of Total Antioxidant Capacity**

A 0.11 M stock solution of DPPH was prepared before each analysis by dissolving the adequate quantity of DPPH in methanol. To a centrifuge tube protected against light by aluminum foil were added 0.1 mL of the diluted extract and 4 mL of DPPH solution, and after 10 min, the absorbance of the mixture was measured at 517 nm against a blank. The free radical scavenging activity of each extract, expressed as a percentage, was calculated from the formula: A_0_ – A_S_/A_0_ × 100, where A_0_ is the absorbance of the control solution including 4 mL of DPPH, and A_S_ is the absorbance of the DPPH solution including the plant extract.

The FRAP reagent was prepared by mixing 300 mmol/L acetate buffer at pH 3.6, 10 mmol/L 2,4,6-tris(2-pyridyl)-s-triazine (TPTZ) solution in 40 mmol/L hydrochloric acid and 20 mmol/L iron (III) chloride hexahydrate solution at a ratio of 10:1:1 (v:v:v). The reagent was prepared fresh daily and was warmed at 37°C for 10 min in a water bath before use. Then, 0.1 mL of diluted extract was added into 1.9 mL of FRAP reagent, and the solution was diluted to 10 mL with redistilled water. After incubation at 37°C for 10 min in a water bath, the absorbance of the solution was measured at 593 nm.
