# Supplementary material for: Total Phenolic Contents and Antioxidant Potential of Herbs Used for Medical and Culinary Purposes
Source: Plant Foods Hum Nutr. 2018 Oct 29;74(1):61–7. doi: 10.1007/s11130-018-0699-5 (PMC6422988; doi:10.1007/s11130-018-0699-5)
Supplement: Supplementary file 2 — (DOCX 24 kb) [file 11130_2018_699_MOESM2_ESM.docx]

Total phenolic contents and antioxidant potential of herbs used for medical and culinary purposes. Plant Foods for Human Nutrition.

Beata Ulewicz-Magulska · Marek Wesolowski, Department of Analytical Chemistry, Medical University of Gdansk, Gen. J. Hallera 107, 80-416 Gdansk, Poland, *E-mail address*: [marwes@gumed.edu.pl](mailto:marwes@gumed.edu.pl)

**Table 1** Chemical characteristic of the herbs and spices under study

| Sample number | Herbs and spices | Plants species | Principal chemical constituents | Ref. |
| --- | --- | --- | --- | --- |
| Medicinal herbs from Lamiaceae family | | | | |
| 1 | Rosemary leaves | *Rosmarinus officinalis* L. | 1-2.5% of essential oil (1,8-cineole, (+)-borneol, camphor, α- and β-pinene), phenolic acids (rosmarinic, caffeic, syringic, chlorogenic, ferulic), flavonoids (luteolin, diosmetin, quercetin), bitter diterpenic compounds, triterpenes, steroids, lipids | [1-4,6] |
| 2 | Sage leaves | *Salvia officinalis* L. | 1-2.5% of essential oil (thujone, cineole, camphor, borneol, pinen), bitter diterpenic lactones, tannins, flavonoids (quercetin, luteolin and apigenin derivatives) | [1-3] |
| 3 | Thyme herbs | *Thymus vulgaris* L. | 0.5-2.5% of essential oil (thymol, carvacrol), tannins, phenolic acids (rosmarinic, caffeic, chlorogenic), triterpenic acids, flavonoids (quercetin, luteolin) | [1-3,4] |
| 4 | Oregano herbs | *Origanum vulgare* L. | 0.12-1.2% of essential oil (carvacrol, thymol), flavonoids (luteolin, diosmetin and apigenin derivatives, quercetin,), phenolic acids (rosmarinic, caffeic, chlorogenic) | [2-4] |
| 5 | Basil herbs | *Ocimum basilicum* L. | 0.04-0.07% of essential oil (linalool, camphor, α-pinene, eugenol), tannins, flavonoids (quercetin and kaempferol glycosides) | [1,3] |
| 6 | Melissa leaves | *Melissa officinalis* L. | 0.02-0.2% of essential oil (citral A and B, citronellal), phenolic acids (rosmarinic, caffeic, ferulic, chlorogenic), triterpenic acids, flavonoids (luteolin and apigenin derivatives, quercetin), essential metals, tannins, triterpenes | [1,3,5] |
| 7 | Peppermint leaves | *Mentha piperita* L. | 0.5-4.0% of essential oil (menthol, esters of menthol), tannins (6-12%), phenolic acids (rosmarinic, caffeic), flavonoids (luteolin, diosmetin, apigenin and their glycosides) | [1,3] |
| Medicinal herbs from Apiaceae family | | | | |
| 8 | Caraway seed | *Carum carvi* L. | 3-7% of essential oil (D(+)-carvone, D(–)-limonene, α- and β-pinene), phenolic acids (caffeic), flavonoids (kaempferol, quercetin glycosides), proteins, fatty oils, carbohydrates | [1-3] |
| 9 | Lovage roots | *Levisticum officinalis* Koch. | 0.4-1.7% of essential oil (phthalides, α- and β-pinene, esters of acetic and valeric acids), cumarins, phenolic acids (chlorogenic, caffeic), starch | [1,3] |
| 10 | Angelica roots | *Archangelica officinalis* Haffm. | 0.35-1.5% of essential oil (α-pinene tannins, flavonoids (naringin derivatives) | [1,3] |
| Spices from Lamiaceae family | | | | |
| 11 | Rosemary | *Rosmarinus officinalis* L. | 1-2.5% of essential oil (1,8-cineole, (+)-borneol, camphor, α- and β-pinene), phenolic acids (rosmarinic, caffeic, syringic, chlorogenic, ferulic), flavonoids (luteolin, diosmetin, quercetin), bitter diterpenic compounds, triterpenes, steroids, lipids | [3] |
| 12 | Sage | *Salvia officinalis* L. | 1-2.5% of essential oil (thujone, cineole, camphor, borneol, pinen), bitter diterpenic lactones, tannins, flavonoids (quercetin, luteolin and apigenin derivatives) | [3] |
| 13 | Thyme | *Thymus vulgaris* L. | 0.5-2.5% of essential oil (thymol, carvacrol), tannins, phenolic acids (rosmarinic, caffeic, chlorogenic), triterpenic acids, flavonoids (quercetin, luteolin) | [1-4] |
| 14 | Oregano | *Origanum vulgare* L. | 0.12-1.2% of essential oil (carvacrol, thymol), flavonoids (luteolin, diosmetin and apigenin derivatives, quercetin), phenolic acids (rosmarinic, caffeic, chlorogenic) | [2-4] |
| 15 | Basil | *Ocimum basilicum* L. | 0.04-0.07% of essential oil (linalool, camphor, α-pinene, eugenol), tannins, flavonoids (quercetin and kaempferol glycosides) | [3] |
| 16 | Marjoram | *Origanum majorana* L. | essential oil (α-terpineol, linalool), tannins (10%), flavonoids (glycosides of apigenin, diosmetin) | [2,3] |
| 17 | Savory | *Satureja hortensis* L. | 0.5% of essential oil (carvacrol, thymol) | [3] |
| 18 | Hyssop | *Hyssopus officinalis* L. | 1% of essential oil (pinene, pinocamphene), flavonoids, rosmarinic acid, di- and triterpenes | [2,3] |
| Spices from Apiaceae family | | | | |
| 19 | Caraway | *Carum carvi* L. | 3-7% of essential oil (D(+)-carvone, D(–)-limonene, α- and β-pinene), phenolic acids (caffeic), flavonoids (kaempferol, quercetin glycosides), proteins, fatty oils, carbohydrates | [1-3] |
| 20 | Lovage | *Levisticum officinalis* Koch. | 0.4-1.7% of essential oil (phthalides, α- and β-pinene, esters of acetic and valeric acids), cumarins, phenolic acids (chlorogenic, caffeic), starch | [1,3] |
| Spices from Asteraceae family | | | | |
| 21 | Tarragon | *Artemisia dracunculus* L. | essential oil, flavonoids, fitosterols, tannins, vitamins A and C | [3] |

1. Wichtl M (2004) Herbal drugs and phytopharmaceuticals. A handbook for practice on a scientific basis (3rd ed.). Boca Ratton: CRC Press)
2. Bruneton J (1999) Pharmacognosy, Phytochemistry, Medicinal Plants (2nd ed.). New York: Lavoisier Publishing Inc.
3. Kohlmünzer S (2003) Pharmacognosy (in Polish) (5th ed.). Warsaw: PZWL
4. Vallverdu-Queralt A, Regueiro J, Martinez-Huelamo M, Alvarenga JFR, Leal LN, Lamuela-Raventos RM (2014) A comprehensive study on the phenolic profile of widely used culinary herbs and spices: rosemary, thyme, oregano, cinnamon, cumin, and bay. Food Chem 154:299-307
5. Barros L, Duenas M, Dias MI, Sousa MJ, Santos-Buelga C, Ferreira ICFR (2013) Phenolic profiles of cultivated, *in vitro* cultured and commercial samples of *Melissa officinalis* L. infusions. Food Chem 136:1-8
6. Erkan N, Ayranci G, Ayranci E (2008) Antioxidant activities of rosemary (*Rosmarinus officinalis* L.) extract, blackseed (*Nigella sativa* L.) essential oil, carnosic acid, rosmarinic acid and sesamol. Food Chem 110:76-82
